# Improving access and uptake of lung cancer screening with a focus on health inequity: the views of professionals involved in the UK NHS lung cancer screening programme

**DOI:** 10.1186/s12885-026-15766-0

**Published:** 2026-03-09

**Authors:** Arbaz Kapadi, Gemma Howard, Zoe Merchant, Philip A J Crosbie, David P French, Lorna McWilliams

**Affiliations:** 1 Manchester Centre for Health Psychology, Division of Psychology and Mental Health, School of Health Sciences, Faculty of Biology, Medicine and Health, The University of Manchester, Manchester, UK; 2 North West Lung Centre, Wythenshawe Hospital, Manchester University NHS Foundation Trust, Manchester, UK; 3 Division of Immunology, Immunity to Infection and Respiratory Medicine, School of Biological Sciences, Faculty of Biology, Medicine and Health, The University of Manchester, Manchester, UK

**Keywords:** Lung cancer, Screening, Service design and delivery, Participation, Health inequity

## Abstract

**Background:**

There is strong evidence that targeted lung cancer screening is effective, but equitable access remains essential to prevent widening health disparities. This study examines the views of professional stakeholders involved in the design and delivery of the UK NHS Lung Cancer Screening Programme (LCSP) with a particular focus on access, uptake and health inequity.

**Methods:**

Individual semi-structured interviews were conducted with twenty-one professionals involved in the set-up or delivery of LCSP services across England. Data collection took place between May and August 2024. Data were analysed inductively using reflexive thematic analysis.

**Results:**

Professionals’ views centred on three themes: (1) Constructing the screening population: identifying eligible individuals for screening related to the accuracy of primary care data, decisions around individuals’ fitness-to-participate and monitoring population uptake; (2) From invitation to participation: design and delivery of screening could differ across services with implications for understanding requirements of screening, decision-making and participation; (3) Raising the profile of lung cancer screening: greater coordination between local networks, teams and service providers was viewed as essential to support awareness, access and uptake.

**Conclusions:**

Ensuring equitable lung cancer screening access requires coordinated strategies across the screening pathway, including improved data collection, clearer clinical guidance, greater understanding of population engagement and more awareness across stakeholder groups. Addressing equity-related factors is crucial to ensuring access and uptake of the LCSP as it expands into a population-wide programme across England by 2030.

**Supplementary Information:**

The online version contains supplementary material available at 10.1186/s12885-026-15766-0.

## Introduction

### Background

Lung cancer is the leading cause of cancer death globally [1, 2]. Smoking is considered the key risk factor, responsible for approximately 85% of all cases [3]. The high mortality is partly attributed to the asymptomatic nature of early-stage disease, resulting in late diagnoses where curative treatment options are limited [4]. Given the poor prognosis, lung cancer early detection represents a global health ambition [3–5]. International studies have demonstrated the effectiveness of lung cancer screening (LCS) (using low-dose computed tomography (LDCT) scanning), reporting a 20–24% relative reduction in mortality [6–8]. The UK NHS Lung Cancer Screening Programme (LCSP) [9] (previously known as the NHS Targeted Lung Health Check (TLHC) Programme) has been established regionally across England since 2019, having initially been delivered across pilot sites characterised by areas of high smoking rates and high levels of deprivation (specifically areas of socio-economic disadvantage, which includes multiple dimensions of disadvantage including income, employment, education, health and access to services) [10–13]. In 2023, the intention for a population-wide programme across England was officially announced with the aim to reach 100% coverage by March 2030 [14].

One marker indicating success of cancer screening is the need for adequate coverage. Whilst the LCSP is not as established as other programmes, uptake remains low (20.4% − 52.6%) in comparison with bowel (67.6%) and breast (70.7%) cancer screening [12, 15–17]. A scoping review to understand uptake of LCS from the perspective of those invited, reported barriers including fear of cancer, fatalism and mistrust of health professionals [18].

Significant social, ethnic and health inequalities have been reported in lung cancer incidence within the UK, which may be further evident at the point of screening uptake [15, 19, 20]. Smoking rates, for example, are higher in areas of deprivation with people living in these areas twice as likely to develop lung cancer [21]. Yet, screening uptake is lower in socioeconomically deprived groups and people who currently smoke [19, 22–25]. Specifically, deprivation can reflect unequal structural conditions that influence both lung cancer risk and the opportunity to access screening [21, 26, 27]. Moreover, individuals living with comorbidities or disabilities are more common among the screening-eligible population yet evidence from other screening programmes report lower uptake from these groups [28–30]. It is not known whether lung screening professionals expect this to be similar.

It is crucial that access to cancer screening is equitable, such that those at risk, regardless of background, have equal opportunities to access the benefits, without disproportionate barriers to participation. There are, then, additional considerations for uptake of LCS given the targeted eligibility criteria and subsequent demographics of groups accessing screening [31]. The following criteria are currently used to invite individuals to assess for potential screening: (a) aged between 55 and 74 years, 364 days of age (at the date of first LDCT scan), (b) registered with a GP practice, (c) have ever smoked [9]. Individuals considered high risk, as measured by the Prostate, Lung, Colorectal and Ovarian Cancer Screening Trial Modified 2012 (PLCOm2012) risk model and the Liverpool Lung Project (LLPv2) risk model, will be offered an LDCT scan (See Appendix 1 for entire LCSP pathway). Exclusion criteria for screening includes: (a) weight or physical size exceeding restrictions for use of the scanner (> 200Kg), (b) participant unable to lie flat on the scanner, (c) poor physical fitness such that treatment with curative intent would have a negative effect [9].

There is limited evidence capturing the views of professionals involved across LCSP services. A single study reports professionals’ views towards the preparedness of the LCSP; however, this was restricted to one geographical area with little examination of inequities within screening design and delivery [32]. Furthermore, whilst the LCSP standard protocol [9] explicitly mentions the need to ensure screening is accessible to all, it is unclear how this is being operationalised in local services. Investigating how to improve access to the LCSP, from the perspective of professionals responsible for implementing strategies, is therefore vital whilst the programme is in its infancy and more amenable to modification.

### Study aim

Our aim was to identify perceived factors related to access to and uptake of screening from the perspective of professionals involved in the design and delivery of LCSP services. This included eliciting views towards current invitation strategies and patient participation, along with identifying strategies to increase screening accessibility.

## Methods

### Design and setting

A cross-sectional qualitative design using semi-structured interviews was employed. The study obtained ethical approval from The University of Manchester Proportionate Research Ethics Committee (UREC) in February 2024. Data collection took place between May and August 2024.

### Participants and procedure

To ensure diversity across roles, involvement and geographical spread, purposive sampling was used to identify professionals involved in either (a) the set-up and implementation of the LCSP, or (b) in LCSP service delivery and/or supporting screening access and uptake. During the study period, the programme had only been partially rolled out across England. The study was advertised in the NHS England LCSP newsletter, whilst an invitational flyer was submitted onto the NHS Futures platform, an online workspace used by healthcare professionals. Further study advertisement included through mailing lists of societies and groups such as The Lung Cancer Policy Network, British Thoracic Oncology Group (BTOG) and The Greater Manchester Learning Disability Cancer Network. Interested professionals were invited to contact the research team through the provided email for further study information. Professionals were also invited directly through email using publicly available contact information e.g. members of the UK National Screening Committee (UK NSC) and LCSP Clinical Expert Advisory Group (CEAG). Snowballing methods were further employed with interviewees suggesting persons of interest. Where identified, participant information sheets were circulated via email to participants by the research team (AK, GH) asking whether they wished to participate in a one-off interview. Participants were offered the opportunity to receive a £30 voucher as reimbursement of their time.

Two topic guides were developed to reflect participant involvement in either set-up or delivery of LCSP services [See Appendix 2 and 3]. Both topic guides shared a similar set of questions regarding invitation strategy, eligibility criteria, and barriers and adjustments to screening access. The topic guide for professionals involved in set-up included an additional set of questions regarding national guidance, screening service capacity and development. Both topic guides were piloted. The topic guide for professionals involved in set-up was piloted with a senior clinician from the CEAG. The topic guide for professionals involved in delivery was piloted with a LCSP manager.

Semi-structured interviews were conducted virtually over Microsoft Teams. Interviews with set-up and implementation professionals were conducted by AK; interviews with delivery professionals were conducted by GH. All participants provided verbal informed consent. Consent was taken on the day with the researcher going through the form with the participant, which had been uploaded onto the screen. Based on verbal agreement, the interview, then, commenced. No participants refused participation, after having initially agreed, and neither did any participants drop out during the interview stage. Demographic and occupational information was also collected from each participant (with consent) for purposes of reporting. The median interview duration was 49 min (range 21–77 min). All interviews were audio-recorded (with consent) and auto-transcribed using the Microsoft Teams function. Transcripts were reviewed against recordings by AK and GH to ensure accuracy. Informed by the concept of information power [33], data collection continued until the research team were satisfied that sufficient data had been collected and no new information was being generated; this was determined by regular debrief discussions between AK, GH and LMcW.

### Analysis

The approach to data analysis was not underpinned by a specific theoretical framework. Instead, data were analysed using reflexive thematic analysis from an essentialist, experiential perspective [34] to explore professional’s views without applying predefined theoretical constructs. This entailed examination of the realities and meanings ascribed by participants as experienced, and through which patterns were identified across the data set [35].

Each transcript was systematically read multiple times for familiarisation before formal coding was undertaken. Reflexive logs, undertaken after each interview to highlight key points of discussion, were also reviewed. Data were coded using NVivo 12, a qualitative data analysis computer software package. To begin the coding process, three earlier transcripts (across set-up and delivery interviews) were coded independently (AK, GH, LMcW) followed by a meeting to discuss views about the data, compare coding and review any particular differences. This was also important to determine the breadth of analysis to apply. AK undertook coding of all transcripts. Coding was conducted at an inductive-manifest level, whereby codes were derived from the data rather than being guided by preconceived ideas. Coding was iterative with developing codes compared and refined across transcripts, with this shared and discussed in regular face-to-face team meetings. Patterns were identified within the codes and initial themes were created, with these collaboratively refined though discussion between the team (AK, GH, LMcW, DPF). Themes and codes were compared across the data set with analysis concluded when no new codes were generated from the data. Codes, developing themes and the final thematic structure were reviewed and refined by AK, GH, LMcW, DPF and PAJC.

### Reflexivity and positionality

The research team consists of three males and three females. Five team members (GH, LMcW, DPF, ZM, PAJC) identify as White British, whilst AK identifies as Asian British. Four team members have health psychology backgrounds (across masters, doctorate and professorial levels). ZM is an occupational therapist who has experience of managing a LCSP service and is a member of the LCSP Effectiveness Standards Task and Finish Group. PAJC is a lung cancer clinician and a member of the CEAG. Both ZM and PAJC have been involved in organising and leading studies that investigate LCS. Likewise, LMcW and DPF have conducted several studies across other national cancer screening programmes, with a focus on early detection, access and health inequity. Subsequently, this research study was approached with prior interest and knowledge of health inequity in screening, along with expertise in qualitative health research and methodology.

## Results

In total, twenty-one interviews were conducted (See Table 1).

**Table 1:** Demographic and occupational characteristics of interviewees

Characteristic	*N*
**Sex**	
Male	8
Female	13
**Age Range (years)**	
18–30	1
31–40	6
41–50	6
51–60	6
61–70	2
Primary Job Role*	
Clinician (inc. general practitioners, nurses, respiratory consultants, cancer clinicians and leads)	12
Health Inequalities and Outreach (inc. disabilities lead, lung cancer charity health representative, community and engagement officers)	4
UK NHS Lung Cancer Screening Programme (LCSP) Project Management	5
**Cancer Alliance**	
Northern	1
Greater Manchester	2
Cheshire and Merseyside	1
West Yorkshire and Harrogate	1
South Yorkshire and Bassetlaw	2
West Midlands	3
East Midlands	1
East of England (North)	3
East of England (South)	3
Thames Valley	1
North Central London	1
Surrey and Sussex	1
Somerset, Wiltshire and Avon	1

*Six interviewees were also members of the LCSP Clinical Expert Advisory Group (CEAG); an independent group providing expert advice on clinical issues in lung cancer, including informing the LCSP standard protocol

**Fig. 1:**
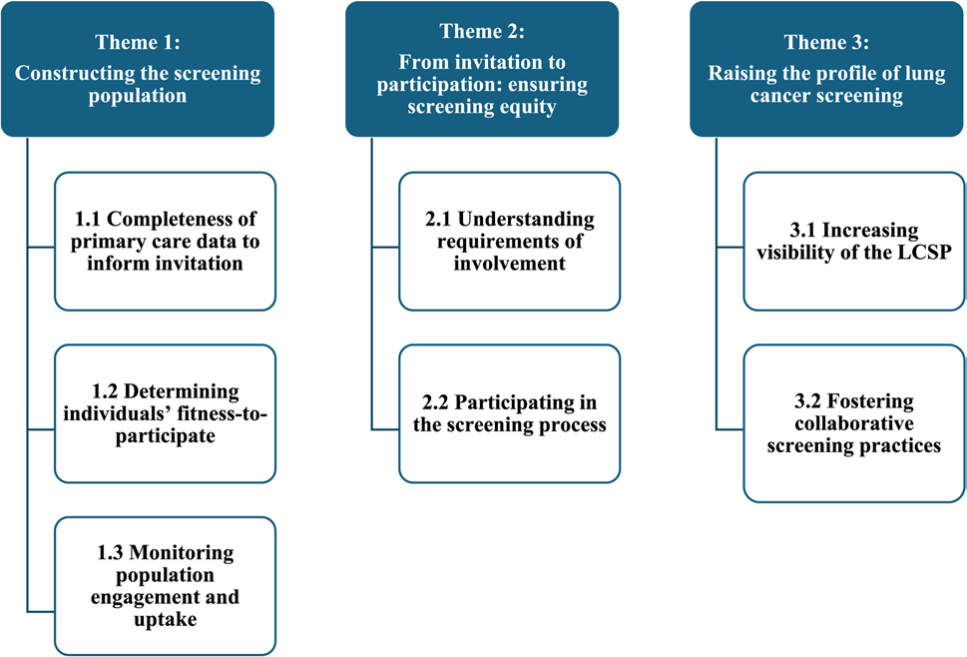
Thematic structure

There was recognition that the LCSP was operating at different phases across England. With this considered, coding was organised under the following three themes (See Fig. 1): (1) Constructing the screening population, (2) From participation to invitation: ensuring screening equity and 3) Raising the profile of lung cancer screening.

### Theme 1: Constructing the screening population

Interviewees highlighted key considerations relating to the identification of eligible individuals for the LCSP that include quality of data, coordination across data systems and informed clinical judgement.

#### 1.1 Completeness of primary care data to inform invitation

Doubts over the completeness of smoking status, held within primary care records, were raised by several interviewees, despite representing vital information to inform screening invitations. This was attributed to lack of standardised recording, outdated data and accuracy of self-reporting smoking behaviour.

*“The big trouble, I think with smoking status is that, not that it won’t be coded at all, but that our patients will have, I don’t know, 23 different codes for their smoking status because they’ll come in and their GP will ask them about it and they’ll put a different code.” (Clinician, #19)*

Some interviewees further highlighted how cultural or religious stigma around smoking could lead to potential underreporting of smoking behaviour within communities e.g. South Asian communities. Subsequently, individuals may not be identified as eligible for screening, influencing uptake from such groups. Further data gaps could arise from incomplete patient records (incorrect smoking status) where, for example, a significant other registered on behalf of an individual; cited examples included amongst older age groups and non-native English speakers.

To overcome smoking data concerns, interviewees deliberated over whether individuals should be invited based on age criteria alone. One service described how they modified their invitation approach to do this:

*“A second message went to everyone over a certain age and gave that onus on to the patient […]*,* ‘if you’ve ever smoked*,* you might be eligible for a lung health check*,* please contact’.” (Health Inequalities and Outreach*,* #14)*.

Though eligibility solely based on age criteria appeared favourable, interviewees were cautious of the cost-effectiveness and extra capacity required, with future changes requiring national team guidance. To aid identification efforts, suggestions included emphasising the importance of reporting smoking status data and regular confirmation, assisted by using data gathering modes such as the NHS app. The completeness of demographic information in patient records, particularly language, was also raised. Where unlisted, it was not guaranteed that individuals would receive the first invitation in their preferred language, with implications on a person’s understanding of LCS and their decision to engage.

*“If you don’t speak English and you get a letter*,* what do you do? […] GPs do not record languages in a formattable way […] so it’s very difficult to get that through to your provider […] say you knew someone was Polish*,* you only find that out on the first contact.”(Clinician, #8)*

#### 1.2 Determining individuals’ fitness-to-participate

It was explained that participation in the LCSP is premised on an individual’s ability to tolerate invasive testing, treatment related to lung cancer or other incidental findings, with this decision made at the risk assessment phase. Clinician interviewees, who were well informed about the evidence behind risk assessment models, questioned the strength of the current assessment process to determine how beneficial participation was at an individual level.

*“You have these very strict criteria on the LLP*,* PLCO*,* and then this bit afterwards*,* ‘Oh*,* and do you think they’re fit enough to get on a scanner or are they going to be fit enough for treatment down the line like surgical resection?’ and we know that people’s ability to estimate performance status is really poor […] I do wonder if there are people who get excluded who shouldn’t.” (Clinician*,* #21)*.

Individuals living with comorbidities or disabilities could be at particular risk of exclusion due to perceived limited functional capacity and ability to go through the various screening stages. This could also operate in reverse.

*“There needs to be a parameter around gauging someone’s fitness at all ages*,* which is one of the issues in the programme […] someone who’s 70*,* in a wheelchair*,* and on Long Term Oxygen Therapy*,* they’ll still be eligible […] providing you can lie flat for stereotactic radiotherapy you can get radical treatment. I am unconvinced that […] is in their best interests or a good use of limited public funds.” (Clinician*,* #18)*.

Clinician interviewees further emphasised how fitness-to-participate decisions become more complex as an individual gets older given the increased possibility of comorbidities and subsequent impact on the potential benefit to be gained from screening. For some, then, whilst the ethical inferences of withholding screening for older persons (75+) were considered, remaining with the current age eligibility criteria (55–74) was favoured with expectation that this would be continually revisited and driven by evidence as the programme developed.

#### 1.3 Monitoring population engagement and uptake

Some services described work undertaken to better understand population engagement, however the majority voiced difficulties in having capacity to do so. Along with lack of time and resources (e.g. key staff personnel), challenges included the absence of centralised IT support infrastructures and formal data collection, leaving reliance on local, and often disorganised, data systems.

*“We know that uptake has not been great. We don’t know exactly where from if that makes sense. So*,* we know anecdotally we’re not getting smokers coming forward*,* but are they smokers from learning disabilities? […] we don’t know.” (LCSP Project Management*,* #13)*.

In failing to collate key uptake insight, interviewees consciously acknowledged challenges in distinguishing participation rate by specific demographics or terms of engagement (e.g. not engaging at all or non-responders to appointments). This meant teams could struggle to coordinate targeted responses to tackle barriers and increase uptake.

*“I don’t know in our programme whether we’ve been curious about the DNAs [people who do-not-attend] […] because that leads on to identifying that actually it’s people who can’t speak English*,* they were sent very complicated invitation letters which they couldn’t read […] we’re not always resourced to be sufficiently curious about whether people can or can’t engage.” (Clinician*,* #11)*.

Professionals discussed needing the space to focus on population engagement but felt pressured to ‘get the service up and running’ and achieve full area coverage. Subsequently, the level of priority or expectation of services to monitor population engagement, as reflected in the availability of resources and support, was unclear.

*“It’s a bit of a juxtaposition […] so the national team’s priority is getting invites sent out and seeing as many people as we can because ultimately payments are generated when we do a lung health check. But then you get that low hanging fruit, don’t you? and you’re not really pooling your resources into looking at probably your most deprived communities because they are more difficult to engage and it’s going to take more to get them through the doors.” (LCSP Project Management, #15)*

### Theme 2: From invitation to participation: ensuring screening equity

The mode and design of invitations were perceived to have a significant impact on invitees’ decision-making regarding access to LCSP services. Interviewees reflected on accessibility at the appointment and scanning stages of the screening pathway, with recognition that this should be ongoing priority.

#### 2.1 Understanding requirements of involvement

Across services, letter invitations (containing an information pack) were predominantly used, with text messaging also used by some to send or follow-up on invitations. However, shared concerns were expressed over the accessibility of invitational materials with implications on invitees’ understanding and ability to make informed decisions to engage with LCS. Possible inequalities introduced at the invitation stage revolved around literacy, language and use of technology.

*“The letter itself is quite complicated, it doesn’t meet accessible information standards either […] The literacy levels in this region are particularly low, so one of my worries is that, right from the point of invite, people don’t understand what they are being invited to.” (Health Inequalities and Outreach, #1)*

To counter potential language challenges *(as discussed in 1.1)*, some services provided QR codes with invitations to allow individuals to access them in their preferred language. Though recognised as a positive facilitator, this required access to a digital device and technological competency; older age groups, or those living with learning disabilities were highlighted as being potentially disadvantaged by this.

Whilst professionals in some services reported providing easy-read invitations with the offer of a phone call discussion, others reflected on their approach as potentially being ‘reactive’ with individuals required to seek out adjusted versions of documents. This was subsequently recognised as a serious risk to engagement.

*“I work with the GP practices […] and try and tailor the information, but our invite letters go straight from a central hub, all sort of batched and gone. […] so having to then go looking for the easy-read information or the videos that give you that step-by-step walkthrough [of the screening process] […] have you got that ability to go looking for it?” (Health Inequalities and Outreach, #9)*

#### 2.2 Participating in the screening process

Opt-out invitations, followed up with reminders, were perceived to be better for participation as this provided a timed risk assessment appointment. This said, some expressed concern that the appointment link could have short expiration (within a week to fortnight in examples given). More broadly, service capacity and IT infrastructure could dictate invitation strategy, at times, to the possible detriment of greater participation.

*“We’re doing opt-in mainly because of logistics […] opt-out…you need a big team or a lot of capacity to do it. It also makes bookings quite tricky because […] you overbook your slots because you know a certain percentage will and won’t attend […] In an ideal world*,* opt-out is the way to go*,* but we all know what the state of the NHS is these days*,* resources are limited.” (LCSP Project Management*,* #4)*.

Telephone-based risk assessments, used by the majority, were viewed positively as they minimised attendee travel requirements. However, they could equally present disadvantages for individuals with hearing or visual impairments and those requiring carer or interpreter support. In-person appointments could be offered and extended in duration (20 to 40 min) to accommodate support needs. Although, the individual may need to request this first, adding an additional step to participation.

*“It’s probably not as an accessible service as we would like yet […] I am aware that if someone’s hard of hearing or deaf, if they want to request an appointment or change their appointment, the option is telephone, which is obviously no good […] the majority of our appointments are done on the phone and there’s very limited face-to-face appointments.” (Health Inequalities and Outreach, #9)*

Mobile community truck sites were considered beneficial for bringing screening services closer to individuals. Equally, potential sensory issues were highlighted at some mobile delivery sites and whilst it appeared that most offered wheelchair access, an example was given where this was absent. Furthermore, logistical issues (e.g. generator failure) could result in mobile facilities closing until resolved, requiring rescheduling of appointments and delaying participation.

*“The mobile unit does not have wheelchair access*,* which to me is incredible […] there is a very loud generator outside*,* and when I say loud*,* it’s a really distracting noise. The mobile unit is very white*,* with stripped lighting […] so I would absolutely think*,* at one point*,* we would need the view of a person with autism*,* view of what we can do differently for that group of people” (Health Inequalities and Outreach*,* #1)*.

If preferred, and where possible, individuals could be directed to a fixed screening site (e.g. hospital unit), representing a positive pathway adjustment; some examples of arranging travel were given. Equally, there could be resource and cost implications such as lack of free parking at a hospital site that could pose a barrier.

### Theme 3: Raising the profile of lung cancer screening

Interviewees described the need to strengthen the profile of the LCSP across communities, professionals and services with the aim of raising awareness, facilitating support for access and increasing uptake.

#### 3.1 Increasing visibility of the LCSP

There was consensus for the need to intensify local and national visibility of the LCSP, with campaigns mirroring that of more established national cancer screening programmes. A key conduit for increasing visibility, alongside mass media campaigns, was to involve local groups and charities in co-producing awareness and communication strategies, that could be culturally sensitive and tailored to the demographics of respective areas.

*“People know there’s a breast cancer screening programme*,* there’s a bowel cancer screening programme. They don’t really know there’s a lung cancer screening programme*,* but they ought to […] the main thing is to make it more visible and popular*,* particularly involving the communities where the people might be at risk.” (Clinician*,* #12)*.

Involving community champions was considered crucial to engage with ‘high-risk’ groups or groups traditionally not accessing health services, for reasons, that may include distrust of services. It was further highlighted that local groups and charities may be carrying out existing work to tackle smoking and cancer stigma, and uptake of health services, therefore are already well positioned to address engagement barriers.

*“Finding key people […] so they can educate their groups so if people within those communities have got any concerns*,* they have somebody they can speak to*,* and we can link in those people into hospitals […] narrow that gap because I think sometimes you [as a professional] can only educate somebody to a certain degree” (Clinician*,* #2)*.

‘Myth-busting’ the screening pathway (e.g. moving through the scanner) and positive, non-judgemental messaging was considered key to alleviate potential concerns, with LCSP communication and engagement teams important to this. Some first-hand examples of their outreach work were given such as speaking at faith congregations, visiting prisons and stalls at football matches. However, it was unclear whether each screening service had this capacity or resource available to them, with smaller screening teams seemingly more affected.

#### 3.2 Fostering collaborative screening practices

Interviewees also emphasised a need to strengthen awareness and understanding of the LCSP across professionals and services. Primary care, for example, was identified as a point where engagement efforts could be pivotal, but where awareness of the LCSP could vary. A lack of clarity could potentially impact discussions between professionals and patients regarding screening and being directed to the correct support channels.

*“We’d have patients phoning up their GPs saying*,* ‘I’ve had this letter*,* what’s it mean?’ and then the receptionists or whatever*,* are like*,* ‘oh*,* no*,* I’ve not heard of that.’ And you’d be like ‘no!’. Then you go to that GP and pull up banners in the reception and you’re like ‘look at that*,* it’s there!’ so*,* you know*,* frustrations.” (Health Inequalities and Outreach*,* #13)*.

General LCS awareness amongst professionals could allow for more opportunistic discussion with individuals during care encounters. Furthermore, coordinated efforts between services, across the pathway, was considered crucial to utilise existing resources, streamline the process and reduce the number of people lost at various stages of the screening process. Suggestions included working more closely with care navigators to facilitate engagement in bespoke ways that could prioritise reasonable adjustments e.g. working with inequality teams and occupational therapists to desensitise screening environments. This could reduce anxiety for individuals navigating the screening process, thereby improving both uptake and experience. Overarchingly however, there appeared to be broader messaging around the priority assigned to making participation in screening easier, reflected in the allocation of support, resources and finance.

*“It takes little bit more time to consider reasonable adjustments […] but*,* actually to practice in a different way is harmful […] If we are serious about it being a priority*,* then we need to give it our attention and we need to fund it*,* and it is expensive […] so how serious are we about this?” (Clinician*,* #19)*.

The diversity across sites in respect to invitation strategies and method of programme delivery was considered important to tailor screening to local contexts. At the same time, there was acute recognition of some areas ‘performing better’ than others. Though it was recognised that some services were more established than others, factors such as team size and resources were reflected upon in respect to programme effectiveness. Nonetheless, the impact of this would be felt by individuals living in areas where there was less capacity to tailor strategies or conduct engagement work that identified barriers to uptake.

## Discussion

The findings from this study reveal key insight into the views of professionals, involved in the design and delivery of the LCSP, towards access and uptake factors related to LCS. Theme 1 explored how identification and monitoring of the screening-eligible population rely upon quality of data, coordination across data systems and informed clinical judgement. Theme 2 highlighted how service design and delivery may influence understanding and participation. Theme 3 considered how awareness of the LCSP needs to be strengthened to raise understanding across communities, professionals and services.

Identification of screening-eligible individuals, which is reliant upon access to good quality smoking data, is paramount for screening programme effectiveness [16]. Yet, as reported in our study, concerns remain over smoking data quality with impact on potential identification for LCS. This has been reported elsewhere [36, 37], with data inconsistencies arising due to how questions over smoking status are asked, understood and recorded; this is despite, in the UK (excluding Scotland), a large majority of primary care practices being incentivised and mandated to collect smoking data (through the Quality and Outcomes Framework (QOF)) [38]. The accuracy of self-reported smoking behaviour is also a contributing factor to data inconsistencies, with further implications for screening identification. Stigma around smoking (e.g. negative social judgement) may result in non-disclosure of smoking behaviours [20, 39]. This can be pronounced in certain cultural and religious communities, where tobacco use is prohibited or seen as unconservative practice [40, 41]. Research reports how deprivation, age and ethnicity inequalities are evident in the quality of smoking data [42, 43]. If left unaddressed, this risks furthering disparities in screening participation [16, 44].

Individuals within the age criteria (55–74) and who have ever smoked are considered eligible for the LCSP, with these combined with other factors in an individualised lung cancer risk assessment [9]. This moves beyond age-and-pack-year criteria as used in some countries’ programmes e.g. the US programme is recommended for adults aged 50 to 80 with a 20 pack-year smoking history and who currently smoke or have quit within the past 15 years [45]. Evidence suggests individualised risk assessment for LCS is superior to age-and-pack-year criteria [46, 47]. However, effectiveness is still reliant upon accurate collection of smoking data in the first instance. Moreover, the definition of an ever smoker, which encompasses both people who currently smoke and those who previously smoked, can be ambiguous, and may not necessarily capture recency or frequency of smoking [48]. Screening invitations based on age criteria alone may counter challenges presented by inaccurate smoking data. However, there are additional cost and resource implications to this given more individuals are likely to be invited, of whom many would be ineligible for screening. No national LCSP (as of 2025) has adopted age-only eligibility criteria although this may be a point of future exploration; in particular, opportunities for self or physician-referral outside set eligibility criteria [16, 49]. Relatedly, a recent revision to the UK LCSP standard protocol [9] outlines an aim for individuals within the age criteria (55–74) to have the opportunity to participate, irrespective of smoking history, by the time the programme completes national rollout.

The reported vagueness around fitness-to-participate guidance resonates with findings from Almatrafi et al. [50], who note how identifying and excluding candidates likely to not benefit from LCS continues to be a challenging area. Therein lies a trade-off between gains and harms from screening, with sensitive decision-making required that also considers individuals’ preferences [51]. Reflecting upon interviewees’ suggestions for more nuanced parameters is suggestive of more refined or additional metrics to assess potential screening benefit. Current risk scores (PLCOm2012 and LLPv2) have been shown to select older, more comorbid participants who potentially have fewer life-years to be gained by screening [52]. To this, a more comprehensive frailty metric, which is currently integrated into all primary care systems in England and therefore readily accessible, has been proposed pending further investigation [50, 53, 54]; lung cancer risk factors overlap with comorbid diseases thereby highlighting the significance of frailty [50]. There are also cost-effectiveness implications for more accurate decision-making, leaving resources to be best optimised elsewhere [16, 55].

Good quality communication is crucial to help individuals make an informed decision on whether to access screening. However, it is also where inequities such as literacy, language and technology exist. One study [56] which reviewed an early iteration of the UK LCSP information leaflet (sent with invitations) reported how individuals struggled with understanding information regarding eligibility criteria, abnormal results and incidental findings. Individuals also suggested some form of interpersonal communication to overcome anticipatory anxiety evoked because of potential diagnoses. The role of digital aids for improved communication was raised in our study. However, it is important to recognise how digital access and competency correlate with socioeconomic status, disproportionately affecting vulnerable populations, including racial and ethnic minorities, older adults, and those with disabilities [57, 58]. Therefore, whilst digital tools (e.g. QR codes for translated health information) have had a positive impact [59], alternative support should be readily available (e.g. physical translated copies for participants for whom adjustment to online provision is not appropriate).

Understanding engagement across different groups to facilitate strategies for access is crucial given known inequities in cancer screening uptake [19, 23, 31, 60]. In our study, a focus on uptake appeared to be prioritised over equity, with several professionals citing pressures of rolling out screening in the first instance. Furthermore, our study findings suggest how services’ responses could be reactive, with onus placed on individuals to request accessible communications or alternative modes of engagement. Evidence documenting the uptake of health services has shown how some individuals can find it difficult to articulate the adjustments they need due to their health situation (e.g. learning disabilities, cognitive impairments) [61, 62], whilst others may feel they are being demanding, discouraging them to seek assistance [63]. Indeed, O’Dowd et al. [16] highlight how shared decision-making is crucial to optimising equitable participation, whilst supportive infrastructures (i.e. resources, strategy and team capacity) are important to instigate mechanisms for local evaluation, innovation and research in the LCSP. Learning may be drawn from the more established breast and bowel national cancer screening programmes, where providers are advised to use screening health equity audits, to review and address inequalities in uptake [64, 65]. The LCSP equality impact assessment [31] can also be a useful resource for local services to help reflect upon practice.

As per national programme guidance [66], services decide upon respective strategies (invitation, appointment and screening modes) to reflect local context, capacity and access to resources. Several services, for example, use mobile screening units which are recognised as positive facilitators for equitable engagement by bringing care closer to communities [13, 67, 68]. Equally, mobile sites are not immune to physical and logistical obstacles [69], requiring services to offer alternatives such as accessing screening at hospital sites. Research [70–72] indicates that an opt-out invitation to a timed appointment results in higher attendance, whilst reminder and re-invitation strategies have shown to improve participation among groups less likely to respond to the first invitation (e.g. people who currently smoke, greater socio-economic deprived communities, minority-ethnic groups) [12, 60]. Whilst interviewees in our study generally shared this view of opt-out invitations improving overall uptake, they did not necessarily reflect upon how different invitation strategies could improve uptake from particular groups. In doing so, there is a potential failure to understand the opportunities and challenges to accessing screening for different groups. Furthermore, reflecting on the diversity of strategies employed, it is possible that quality of screening services may become highly dependent on geographical location or the specific service provider, leading to inequitable experiences for individuals with similar needs; this is despite each screening service receiving the same financial allocation from the national team. There is also a challenge to evaluate the effectiveness and efficiency of screening fairly if respective programmes are operating to different standards.

A key study finding was to accelerate awareness of the LCSP amongst different stakeholder groups. Awareness of LCS continues to be a key priority for many countries where programmes are being delivered [22, 73, 74]. Further impetus is required to establish and maintain local networks (e.g. with community leaders and champions) to help co-produce communications and relatable messaging, which includes tackling lung cancer misconceptions and stigma. The link between lung cancer stigma and individuals failing to seek medical help and support is well-established [75, 76]. Additionally, greater coordination between teams and services is also likely to help with informed decision-making and facilitating supportive engagement where necessary. Recommendation and endorsement across services such as primary care, and community networks have been highlighted as key factors for engagement across screening programmes [16].

### Strengths and limitations

This study addresses a key gap in the literature by gathering the views of professionals involved in the design and delivery of the UK LCSP, which is, otherwise, limited. Understanding professionals’ views are vital as they are responsible for facilitating strategies for the uptake of screening. Further strengths include the use of a qualitative, interview approach with a diverse range of professionals, involved across set-up (including CEAG members who contribute toward national level guidance), local delivery and in supporting access to screening (inequality and outreach leads). Furthermore, thirteen cancer alliances, spread across areas of England, were represented in the interview cohort.

A potential limitation is the fewer number of interviews conducted with professionals involved in delivering screening; they are likely to have greater day-to-day interactions with individuals and thus knowledgeable about the factors influencing access. Furthermore, professionals’ experiences are based on the current phase of their respective screening service. Subsequently, we do not claim the study to be an evaluation of the UK LCSP, but rather, a snapshot, at a point of time, of key considerations as raised by those involved. As the UK LCSP is continually maturing, it is possible that some of the suggestions discussed in this manuscript may have been adopted in practice since the study was conducted. We further stress that the findings presented in this article are participants’ views and the authors’ interpretation of them.

### Implications for practice and research

There is a need to better understand factors influencing the described disparities in data accuracy. Allocating sufficient resources to, and the development of more accessible approaches for data collection and efficient electronic systems for data entry, is crucial to improve the completeness of smoking data. This involves more regular and opportunistic confirmation of smoking status, achieved through, for example, text alerts and encouraging people to update smoking status using the NHS health app; this has also been suggested elsewhere [16, 24].

The public hold a key role to disclose their smoking behaviour accurately. Subsequently, services and healthcare professionals have responsibilities to educate them on the importance of collecting this data and encourage data sharing with assurances of data protection; localised efforts and awareness initiatives may help with this. In respect to screening criteria and assessment, clearer guidance for assessing individuals’ fitness-to-participate is important to ensure that screening is accessed by those where most benefit will be gained. Further practice-informed research such as the possible refinement or addition of risk assessment metrics (e.g. frailty) [50] may be beneficial here. Any change to screening assessment will naturally require training for professionals.

Screening providers must review methods of invitation and mode of screening with a view to prioritising accessibility, which considers language challenges, diverse health literacy levels and physical access barriers. This will require monitoring population engagement to identify factors associated with non-engagement or drop-off and develop tailored interventions. For this, dedicated staff, and subsequent training, time and resource to analyse data, build relations with communities and co-produce culturally informed communications, is required. An overarching aim should be to raise awareness of the LCSP across communities, professionals and services through mass media campaigns, whilst fostering cross-sectoral partnerships with public health teams, community organisations and local networks. These collaborations can facilitate engagement, enable reasonable adjustments, and create opportunities for discussions about LCS. If primary care is to take a greater role in the LCSP, further research is required to examine the additional burden versus the benefits gained. Overall, the findings from our study provide a foundation for subsequent theory-informed implementation research.

## Conclusion

Given known inequities across the lung cancer care continuum, and evidence from other cancer screening programmes, it is crucial that access to the LCSP is equitable. However, uptake currently remains varied and socially patterned. This study highlights multiple individual and system-level factors located across the LCSP pathway, which require further consideration to ensure equitable engagement can be achieved. Identifying priorities regarding health inequalities within the UK LCSP is important to ensure standardisation, minimise geographical variation and prioritise equity of access as the programme is rolled out to population level by 2030.

## Supplementary Information

Supplementary Material 1.

## Data Availability

The data that support the findings of this study are openly available in Figshare at [10.48420/31211323].
