# Supplementary material for: Improving access and uptake of lung cancer screening with a focus on health inequity: the views of professionals involved in the UK NHS lung cancer screening programme
Source: BMC Cancer. 2026 Mar 9;26:483. doi: 10.1186/s12885-026-15766-0 (PMC13085362; doi:10.1186/s12885-026-15766-0)
Supplement: Supplementary file 1 — Supplementary Material 1. [file 12885_2026_15766_MOESM1_ESM.docx]

**Supplementary Information – Appendices**

**Appendix 1**

**UK NHS Lung Cancer Screening Programme (LCSP) Participant Pathway (adapted from v3.0 LCSP Standard Protocol)**

**
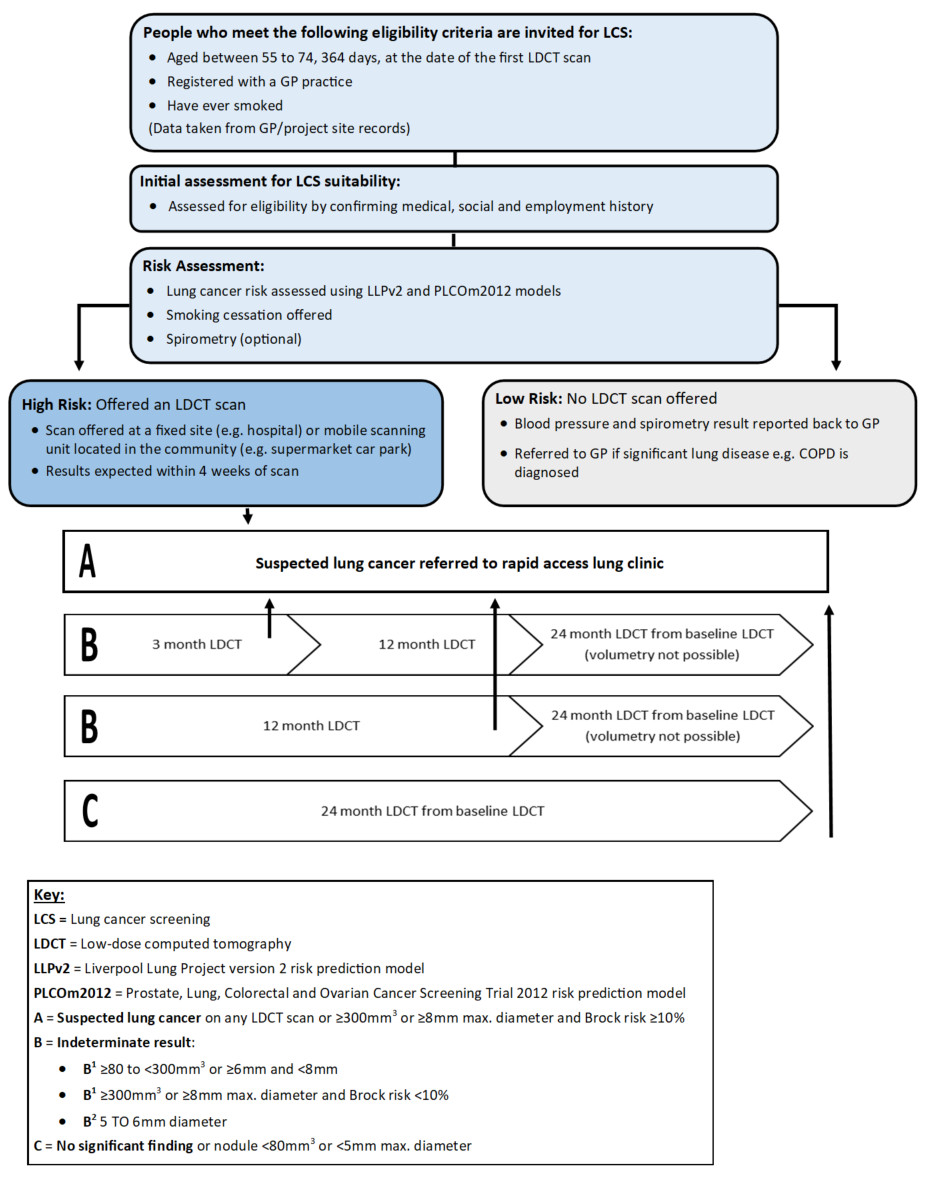
**

**Appendix 2**

**Interview Topic Guide 1: Professionals involved in the set-up and implementation of the UK NHS Lung Cancer Screening Programme (LCSP)**

**Setting up and implementing screening**

- Could you please describe the key considerations for setting up lung cancer screening (LCS)/local service in your area?
- How does the site/programme fit in with the timeline of LCSP pilots?
- How did you prepare for the set-up of the programme?
- How does the standard LCSP protocol inform your service?
- What does the evaluation process of your local LCS service look like?
- What are your thoughts on: a) the supporting infrastructure for LCSP rollout, b) education, training and awareness of the LCSP for staff

**Access and uptake of screening**

- Can you tell me about how those eligible to take part in the LCSP are invited within your area? e.g. invitation, assessment, mode of screening
- What do you think about how risk is assessed as part of LCS? What are your thoughts on the evidence base underpinning this?
- What do you think about the factors currently used to determine eligibility to receive an invite? e.g. smoking
- What is the process for reaching out to and inviting individuals to participate in the LCSP?
- What are your thoughts about the uptake from those who have been invited so far?
- Which groups, do you think, may be more likely to experience greater barriers to participating in LCS? and why?
- Are there any targeted approaches being considered to secure access to screening for these groups?
- There is lower uptake to LCS compared to other programmes (like breast cancer). What do you think about this?

**Reasonable adjustments within screening pathway**

- Do you have any thoughts on how health inequalities affect access to and uptake of LCS?
- Have you implemented any reasonable adjustments within the design of LCS? How?
- Can you tell me about any experience(s) you have had helping someone decide about whether to attend screening? (if you have no experience, ask how they would go about helping someone make a decision and why they would use that approach)
- What are your thoughts on including the presence of a) certain comorbidities and b) disabilities as inclusion criteria for LCS?
- What are the potential issues associated with screening for patient with a) comorbidities and b) disabilities?
- What do you think would be needed to improve uptake of LCS for: a) the general population, b) patients living with comorbidities, c) patients living with learning disabilities

**Summary of discussion**

- How do you feel the LCSP, in its current design, is meeting its intended aims? a) What do you feel is working well? b) What do you feel is working less well?
- What do you think are the most important things that need to be considered as the LCSP continues to be rolled out?
- Was there anything that you would like to add? or thought we would discuss and have not done today?

**Appendix 3**

**Interview Topic Guide 2: Professionals involved in the delivery of the UK NHS Lung Cancer Screening Programme (LCSP)**

**Access and uptake of screening**

- Can you tell me about how those eligible to take part in the LCSP are invited within your area? e.g. invitation, assessment, mode of screening
- What do you think about how risk is assessed as part of lung cancer screening (LCS)?
- What do you think about the factors currently used to determine eligibility to receive an invite? e.g. smoking
- What is the process for reaching out to and inviting individuals to participate in the screening programme?
- What are your thoughts about the uptake from those who have been invited so far?
- Which groups, do you think, may be more likely to experience greater barriers to participating in LCS? and why?
- Are there any targeted approaches being considered to secure access to screening for these groups?
- There is lower uptake to LCS compared to other programmes (like breast cancer). What do you think about this?

**Reasonable adjustments** **within screening pathway**

- Do you have any thoughts on how health inequalities affect access to and uptake of LCS?
- Have you implemented any reasonable adjustments within the design of LCS? How?
- Can you tell me about any experience(s) you have had helping someone decide about whether to attend screening? (if you have no experience, ask how they would go about helping someone make a decision and why they would use that approach)
- What are your thoughts on including the presence of a) certain comorbidities and b) disabilities as inclusion criteria for LCS?
- What are the potential issues associated with screening for patient with a) comorbidities and b) disabilities?
- What do you think would be needed to a) improve uptake of screening generally, b) patients with comorbidities, c) patients with learning disabilities

**Summary of discussion**

- How do you feel the LCSP, in its current design, is meeting its intended aims? a) What do you feel is working well? b) What do you feel is working less well?
- What do you think are the most important things that need to be considered as the LCSP continues to be rolled out?
- Was there anything that you would like to add? or thought we would discuss and have not done today?
